# Editorial: Use of barley and wheat reference sequences: Downstream applications in breeding, gene isolation, GWAS, and evolution, volume II

**DOI:** 10.3389/fpls.2022.1034744

**Published:** 2022-10-31

**Authors:** Peter M. Dracatos, Hikmet Budak, Pierre Sourdille, Dragan Perovic

**Affiliations:** ^1^ Department of Animal, Plant and Soil Science, La Trobe University, AgriBio, Bundoora, Australia; ^2^ Cereal Genomics and Genome Editing, Montana BioAgriculture, Inc., Missoula, MT, United States; ^3^ UMR 1095 Genetics, Diversity and Ecophysiology of Cereals, 5, Institute for Agriculture, Food and the Environment (INRAE)–Université Clermont-Auvergne, Clermont-Ferrand, France; ^4^ Julius Kuehn Institute, Federal Research Centre for Cultivated Plants, Institute for Resistance Research and Stress Tolerance, Quedlinburg, Germany

**Keywords:** barley, wheat, pan genomics, GWAS, gene isolation, genotype by sequencing

Cereal grains are the most important food source consumed by human beings. Among these, bread wheat is the most widely grown crop in the world, ranking 2^nd^ only to rice from total production point of view, whereas barley is ranked as the 4^th^ most important cereal. The inherent narrow genetic diversity present within modern cereal crops combined with their large complex genomes had previously created a genetic bottleneck hampering breeding progress as well as applying newly developed applications in biotechnology. Improvements to long-read sequencing technologies continue to enhance our ability to generate ultra-contiguous chromosome scale assemblies, thus further improving the efficacy of gene isolation and unravelling the mechanisms of evolution in cereal crop species. Despite the continual decline in sequencing costs and bioinformatic innovation, genotyping by sequencing (GBS) using targeted enrichment protocols and allele re-sequencing is currently the most cost-efficient approach to generate large SNP datasets. This Frontiers in Plant Science research collection comprises 16 articles highlighting the broad utility derived from combining multiple chromosome scale genome reference assemblies with new approaches in quantitative genetics to best exploit favourable genetic trait variation.

Rajendran et al. outlines the different GBS protocols and both their current and future applications established for cereals focussing on barley as a diploid model crop species. The review highlights the utility derived from GBS genotyping approaches to mine global GeneBanks and exploit trait genetic diversity using genome wide association studies (GWAS) and genomic selection (GS).

The recent optimisation of the GBS approach has led to larger scale population genomic approaches in preference to smaller biparental populations to identify the causal genetic variation underlying traits of interest and is largely amenable to complex traits such as yield (Rajendran et al.). Eight studies mainly in wheat used GWAS as an approach to define the genetic architecture of largely uncharacterised complex agronomic traits of interest including nutrient content (Jin et al.; Juliana et al.), herbicide resistance (Kurya et al.), lodging resistance (Bretani et al.), yield related traits (Miao et al.; Sheoran et al.; Yu et al.), flowering time and phenology (Bhati et al., Hu and Zuo 2022) and disease resistance (Juliana et al.; Mehnaz et al.; Wang et al.). Except for flowering time, phenology and molybdenum content, these studies highlighted the complex genetic basis of the agronomic traits assessed and the potential to use the GWAS data for a subsequent GS approach.

Juliana et al. investigated the genetic control of grain zinc and iron content in a large collection of wheat breeding lines (n= 5,585) from CIMMYT. The lines were genotyped using 20,556 GBS markers and evaluated for both zinc and iron content between 2018 and 2021 in Mexico. The study identified 141 marker-trait associations on all 21 chromosomes except for chromosomes 3A and 7D. Among them, 29 markers were associated with both iron and zinc content suggesting a shared mechanistic basis. The complex genetic control of these traits highlights the need for GS to efficiently improve nutrient content in wheat.

Similarly, Jin et al. determined the genetic control of grain molybdenum content in bread wheat using 207 accessions and a set of 224,706 SNP markers from the 660k wheat array. The study identified 77 significantly associated SNPs, 52 of which were detected in at least two datasets and 48 out of the 52 were distributed in a small region of 1.37 Mb located in the distal part of the long arm of chromosome 2A. In the region spanning the 2A QTL three plausible candidates including a molybdate transporter 1;2, molybdate transporter 1;1 and molybdopterin biosynthesis protein CNX1, were identified for further functional analysis.

Kurya et al. identified resistance to metribuzin to improve the productivity of bread wheat grown in dryland regions. The 150 diverse accessions were genotyped using the 50K SNP array and phenotyped by measurement of chlorophyll content relative to the untreated control plants. The analysis identified 19 genomic regions including 10 on chromosome 6A, three on 2B and one on 3A, 5B, 6B 6D, 7A, and 7B, respectively. Using the wheat genome assembly, several candidate genes were identified that were involved in herbicide resistance including cytochrome P450 pathways and ATP Binding Cassette superfamilies.

Bretani et al. developed an image-based analysis protocol to accurately phenotype for culm wall thickness and diameter that facilitated a multi-environment analysis to determine the genetic control of culm morphology and lodging in barley. A collection of 261 barley accessions were genotyped using the 50k iSelect SNP array and phenotyped across seven different environments. Most culm morphology traits were highly heritable (>50%) and affected by several genotype-by-environment interactions. The data highlighted the possibility of improving lodging independently from plant height and identified candidate genes involved in hormone and cell wall related pathways.

Bhati et al. investigated the genetic control of phenology and heading date traits in bread wheat across three representative wheat growing regions in India. A large collection of spring wheat breeding lines (n= 4,680) were phenotyped at multiple locations for days to heading and maturity. The phenotypic variation highlighted the importance of the photoperiod associated gene (*Ppd-B1*) and the *Vrn-B1* gene for adaptation of bread wheat across the different environments.

Sheoran et al. combined a large-scale GWAS and artificial intelligence (AI) with genotype-phenotype networking to understand the complex genetic control of spike fertility in bread wheat. GWAS was performed on 200 diverse wheats using the Breeders’ 35K Axiom Wheat Breeders' Array and multiple years of phenotypic data. The study identified 255 significant marker-trait associations (MTAs) with MTAs on chromosome 3A, 3D, 5B and 6A being the most promising for enhancing spike fertility and grain yield.

Juliana et al. used varying numbers of GBS based markers and performed a large-scale study to identify spot blotch resistance in a panel of 6,736 (separated into seven specific panels) advanced bread wheat breeding lines. Ninety-six significant markers were common amongst the seven panels mapping to chromosomes 1A, 1B, 1D, 2A, 3B, 4A, 5B, 5D, 6B, 7A, 7B, and 7D, and included possible linkage to previously known disease resistance loci including the *Lr46, Sb1, Sb2* and *Sb3* genes. Importantly the study identified favourable alleles for spot blotch resistance on the same 2NS translocation from *Aegilops ventricosa* where two markers were associated with increased grain yield across multiple environments in India and Mexico.

Hu and Zuo combined GWAS and an expression profiling approach to identify a promising candidate for the *Hg1* gene controlling glume pubescence on chromosome 1AS encoding a glycosyltransferase-like ELD1/KOBITO 1 in bread wheat. The candidate was identified using the most recent iteration of the wheat reference genome assembly based on tissue specific expression patterns and functional SNP haplotype analysis. The study demonstrated the importance of utilising the latest reference genome data, expression patterns and GWAS data to clone genes in bread wheat.

The availability of a pan-genome for both bread wheat and barley addresses the most common limiting bottleneck in gene cloning projects which is the over-reliance on both the gene order and representation in the available reference genome. Hussain et al. reviewed the status and future directions of wheat improvement in the pan genomic era with respect to important agronomic traits such as yield, quality and both biotic and abiotic stresses.

The utility of the barley and wheat pan-genome lies in being able to use the information either from the most closely related or directly from the sequenced genotype either carrying or lacking the trait of interest as a reference genome thus limiting the number of SNPs, presence absence variations (PAVs), and inversions. This is especially important to improve the efficiency and accuracy of QTL mapping, fine mapping, identify recombination events and gene annotation.

Two studies in wheat reported on utilising the wheat reference genome to dissect yield related traits using a combination of QTL analysis and bulk segregant exome sequencing (BSE-Seq) and meta-QTL (MQTL) analysis, respectively.

Yu et al. investigated the genetic architecture of yield-related traits (spike compactness and length) using a QTL mapping approach in a recombinant inbred line (RIL) population. Three genomic regions were identified on chromosomes 2A and 2D using BSE-Seq. Subsequent linkage map construction and QTL analysis identified six major QTL across more than four environments explaining 7.00-28.56% of phenotypic variation with LOD values varying from 2.50 to 13.22. Three promising candidates were identified based on genomic and expression data for further functional analysis.

Miao et al. performed QTL analysis for thousand grain weight (TGW) in a bread wheat RIL population and identified 45 TWG QTLs, where 10 loci were highly stable across multiple environments. To refine the relatively large TGW QTL intervals the Chinese Spring reference genome was used to perform a MQTL analysis. A total of 267 previously reported QTLs were consolidated and refined to 67 MQTLs. Importantly, five key core MQTLs were refined to <1cM regions corresponding to <20Mb in Chinese Spring enhancing the prospects of candidate gene discovery, validation, and improved marker design.

In barley, reference genomes have undoubtedly enhanced fine mapping studies, candidate validation based on chromosome scale gene annotation and direct comparison of resistant and susceptible haplotypes.

Mehnaz et al. firstly mapped a novel leaf rust resistance gene from an Israeli landrace (AGG396) to chromosome 2HS at the previously characterised *Rph14* locus in barley. Both medium and high-resolution fine mapping narrowed the genetic interval to 0.7cM (corresponding to a 1.17 Mb physical interval) where two annotated NLR (nucleotide-binding domain leucine-rich repeat) genes were identified using the Morex v2 reference genome and deemed the most promising candidates for *RphAGG396*. A closely linked co-dominant marker was designed for marker assisted selection.

Wang et al. fine mapped the barley mild mosaic virus resistance locus *rym15* located on chromosome 6H. A set of 32 KASP markers designed from the barley 50K Illumina Infinium iSelect SNP array, GBS and WGS data were used as a backbone to construct two high-resolution genetic maps based on the resistant donor Chikurin Ibaraki. The locus was resolved to 0.036 cM in the Chikurin Ibaraki 1 × Uschi cross corresponding to a 281kb physical interval. Pan genomic data for susceptible Igri and Golden Promise was compared to a Pac Bio assembly of Chikurin Ibaraki to determine that only two candidate genes contained functional SNPs between resistant and susceptible lines.

Two studies used next generation sequencing technologies to develop a method to track recombination in barley (Schreiber et al.) and determine the structural variants responsible for increased yield in bread wheat (Makhoul et al.) respectively.

Schreiber et al. devised and optimised a method to accurately identify crossover events in the genomes of diploid homozygous inbred barley lines by combining chemical mutagenesis and low-level whole genome shotgun sequencing. The study determined that low-level EMS treatment induced variants in M_3_ populations and can be used to determine recombination rate and frequency. The efficiency of the study was enhanced by direct comparison of the wild type, Bowman and the near isogenic line carrying a mutation in the *HvMLH3* gene (BW230) which the authors had previously shown to reduce the genome wide recombination by up to 50%.

Makhoul et al. used single molecule sequencing of barcoded long amplicons to assess sequence polymorphisms in the VERNALIZATION1 (*Vrn1*) gene between homoeologous gene copies on chromosomes 5A, 5B and 5D in a panel of 192 winter wheat cultivars. Both haplotypic and structural variations were subsequently associated with economically relevant agronomic traits including yield, nodal root-angle index, and quality related traits. Structural variations and increased copy-number variation were associated with reduced quality and yield. Furthermore, a novel SNP polymorphism within the G-quadruplex region of the promoter of *Vrn1-5A* was associated with deeper roots in winter wheat.

## Future perspectives

Undoubtedly, third generation sequencing technologies and the increased availability of chromosome scale reference genomes has increased the capabilities of crop scientists to better exploit genetic variations in agronomic traits of interest. This is especially true for complex quantitatively inherited traits such as yield and resistance to abiotic and biotic stress that were previously too difficult to accurately resolve using trait specific biparental populations. Subsequent genetic gain is likely to occur by exploiting the large volume of GWAS datasets using advanced breeding tools such as artificial intelligence and developments in GS.

In parallel, the ability to continually improve genome assemblies for wheat and barley accessions carrying the desired allele of interest is leading to an improved understanding of the biological mechanisms controlling traits such as resistance to disease. Despite the availability of first generation pan genomes for both barley ad bread wheat, due to its simpler diploid genetic structure, the current status of barley genomics is further advanced relative to bread wheat. Further genomic and biological advances in bread wheat are likely to come from developing pan genomic information utilising diverse diploid representative accessions from progenitor species. To best exploit pan genomic data, further opportunities also lie in accurately phenotyping the currently sequenced barley and wheat accessions for a wider array of traits.

## Author contributions

PD prepared the first draft of this editorial. All authors contributed to the article and approved the submitted version.

## Funding

The research of PD was supported by La Trobe University and the Humboldt Foundation. The research of DP was supported by the German Federal Ministry of Education and Research, under the grant number 031B0199B, and by the German Federal Ministry of Nutrition and Agriculture under the grant 2818410B18, and the research of BH by the Montana BioAg. Inc., USA. The research of PS is supported by INRA - French National Research Institute for Agriculture, Food and the Environment.

## Conflict of interest

Author HB was employed by Montana BioAgriculture, Inc.

The remaining authors declare that the research was conducted in the absence of any commercial or financial relationships that could be construed as a potential conflict of interest.

## Publisher’s note

All claims expressed in this article are solely those of the authors and do not necessarily represent those of their affiliated organizations, or those of the publisher, the editors and the reviewers. Any product that may be evaluated in this article, or claim that may be made by its manufacturer, is not guaranteed or endorsed by the publisher.

